# Identification of sex differentiation-related microRNA and long non-coding RNA in *Takifugu rubripes* gonads

**DOI:** 10.1038/s41598-021-83891-w

**Published:** 2021-04-02

**Authors:** Hongwei Yan, Qi Liu, Jieming Jiang, Xufang Shen, Lei Zhang, Zhen Yuan, Yumeng Wu, Ying Liu

**Affiliations:** 1 Dalian Ocean University, Dalian, 116023 Liaoning China; 2 Key Laboratory of Environment Controlled Aquaculture, Ministry of Education, Dalian, 116023 China

**Keywords:** Animal physiology, Reproductive biology

## Abstract

Although sex determination and differentiation are key developmental processes in animals, the involvement of non-coding RNA in the regulation of this process is still not clarified. The tiger pufferfish (*Takifugu rubripes*) is one of the most economically important marine cultured species in Asia, but analyses of miRNA and long non-coding RNA (lncRNA) at early sex differentiation stages have not been conducted yet. In our study, high-throughput sequencing technology was used to sequence transcriptome libraries from undifferentiated gonads of *T. rubripes*. In total, 231 (107 conserved, and 124 novel) miRNAs were obtained, while 2774 (523 conserved, and 2251 novel) lncRNAs were identified*.* Of these, several miRNAs and lncRNAs were predicted to be the regulators of the expression of sex-related genes (including fru-miR-15b/*foxl2*, novel-167, novel-318, and novel-538/*dmrt1*, novel-548/*amh*, lnc_000338, lnc_000690, lnc_000370, XLOC_021951, and XR_965485.1/*gsdf*). Analysis of differentially expressed miRNAs and lncRNAs showed that three mature miRNAs up-regulated and five mature miRNAs were down-regulated in male gonads compared to female gonads, while 79 lncRNAs were up-regulated and 51 were down-regulated. These findings could highlight a group of interesting miRNAs and lncRNAs for future studies and may reveal new insights into the function of miRNAs and lncRNAs in sex determination and differentiation.

## Introduction

Non-coding RNAs (ncRNAs) represent a class of RNA molecules that do not transcribe into protein. It has been demonstrated that as versatile regulators of genome stability, defense against foreign genetic elements, and gene expression, ncRNAs have multiple functions in diverse cellular processes^1^. Several kinds of ncRNAs exist, and among the ncRNAs, microRNAs (miRNAs) and long ncRNAs (lncRNAs) have been widely studied. MiRNAs, usually 18–26 nucleotides (nt) in length, are a highly conserved, single-stranded class of short small ncRNAs present in plants, animals, and some eukaryotic viruses. Since the discovery of the first miRNAs two decades ago, there are > 250 other species with miRNAs and, to date over 2500 miRNAs have been discovered in humans^2^. MiRNAs have been indicated to have pervasive roles in the regulation of gene expression and > 60% of human and > 37% of *Drosophila* protein-coding transcripts are conserved miRNA binding sites, through some degree of selective pressure^3,4^. MiRNAs function in the form of an effector complex named RNA-induced silencing complex (RISC), along with Argonaute proteins. The interaction between the miRNA-RISC and target mRNAs can mediate gene silencing by multiple inhibitory mechanisms, such as translational effects, mRNA deadenylation, and mRNA degradation. MiRNA-target recognitions usually occurr through seed-pairing, and miRNA recognition elements or miRNA binding sites are positioned mostly at the 3ʹ untranslated region (3ʹ-UTR) of mRNAs. However, it was also suggested that it occurs at 5ʹ-UTRs and/or in coding regions. The miRNA regulation strength can be affected by the perfect base pairing between the target site and miRNA seed region, and the factors that influence the consequence of hybridization, such as the number of miRNA recognition elements of the same mRNA and their relative position^5^.

LncRNAs are the most heterogeneous class of ncRNAs ranging from 200 to 100,000 nt in length. Abundant evidence showed that lncRNAs could involved in regulating gene expression by a variety of mechanisms such as inhibiting RNA polymerase activity, binding to the promoters of target genes, and degrading target mRNAs^6^. LncRNAs can also act as targets of miRNAs, repressing the interaction between coding genes and miRNAs, or encoding certain miRNAs as precursors^7^. Moreover, it has also been found to compete with miRNAs through interactions with protein coding genes, and miRNAs can reduce the stability of lncRNAs^8–10^.

Sex dimorphism is one of the most pervasive and diverse features in the animal kingdom^11^. MiRNAs and lncRNAs have been demonstrated to play significant roles in sex differentiation, gametogenesis, and sexual reproduction in vertebrates. For example, at the critical time of sex determination of mice, it has been revealed that miR-124 is able to prevent *Sox9* expression in ovarian cells, which is the first evidence that an miRNA is involved in the process of sex determination and gonad development^12^. In chickens, male-biased miR-107 has been shown to mediate the post-transcriptional regulation of estrogen signaling by directly decreasing the expression of nuclear receptor subfamily 5 group A member 1, and its downstream genes, *Cyp19a1a*^13^. In teleosts, such as in Atlantic halibut (*Hippoglossus hippoglossus*)^14^, rainbow trout (*Oncorhynchus mykiss*)^15,16^, yellow catfish (*Pylodictis fulvidraco*)^17^, Nile tilapia (*Oreochromis niloticus*)^18–21^, common carp (*Cyprinus carpio*)^22^, olive flounder (*Paralichthys olivaceus*)^23^, and medaka (*Oryzias melastigma*)^24,25^, sexual dimorphic miRNA expression was detected in gonads during sex differentiation and development. Moreover, some miRNAs were predicted to be target genes related to sex determination and differentiation. In the protogynous hermaphroditic fish *Epinephelus coioides*, *cyp19a1a* expression, and E_2_ levels are necessary for sex reversal, furthermore, a positive feedback loop estradiol-17β/miRNA-26a/*cyp19a1a* involved in the control of E_2_ production^26^. In mammals, lncRNA X-inactive specific transcript is required for silencing of one X-chromosome during development in females^27^. In mice, the lncRNA *Dmrt1-related gene* has been shown to form a trans-splicing RNA isoform with *dmrt1*, which disrupts the coding region and replaces the 3′-UTR of *dmrt1*, resulting in decreased dmrt1 protein level^28^. In the Chinese soft-shell turtle (*Pelodiscus sinensis*), numerous lncRNAs were shown to regulate the expression of *sox9*, *dmrt1*, *sox3*, *sox8* and *cyp19a*^29^.

The tiger pufferfish (*Takifugu rubripes*) is one of the most valuable commercial fish cultured in Asia. On the market, male fish are more highly valued than females as the mature testes of tiger pufferfish are regarded as a delicacy, and all-male stocks are preferred in aquaculture. Therefore, elucidating the mechanism of sex determination and differentiation is necessary in this species. In 2012, a missense single-nucleotide polymorphism (SNP) in the amhr2 gene has been identified as a likely candidate for the master sex determination polymorphism^30^. However, sex determination is a complex process, involving a large network of interactions among genes as well as between environment and genes, and the factors involved in gonadal sex differentiation of such an important fish species have yet to be fully elucidated. Screening the sex-related mRNAs and ncRNAs in the undifferentiated gonads at the critical stage of molecular sex determination might provide new information on the role of ncRNAs in gonadal function and help to clarify the regulatory network during early sex differentiation. In 2018, we reported the dimorphic expression patterns of sex-related mRNAs in the undifferentiated gonad of *T. rubripes* through transcriptome analysis^31^. However, screening the sexual dimorphic expression of miRNAs and lncRNAs at early sex differentiation stages have not been conducted yet. Thus, the present study aimed to identify sex-biased miRNAs and lncRNAs in the undifferentiated gonads, which might provide a basis for a better understanding of the functions of miRNAs and lncRNAs in regulating the sex differentiation process in *T. rubripes*.

## Results

### Characterization of gonadal small RNA of *T. rubripes*

**Figure 1:**
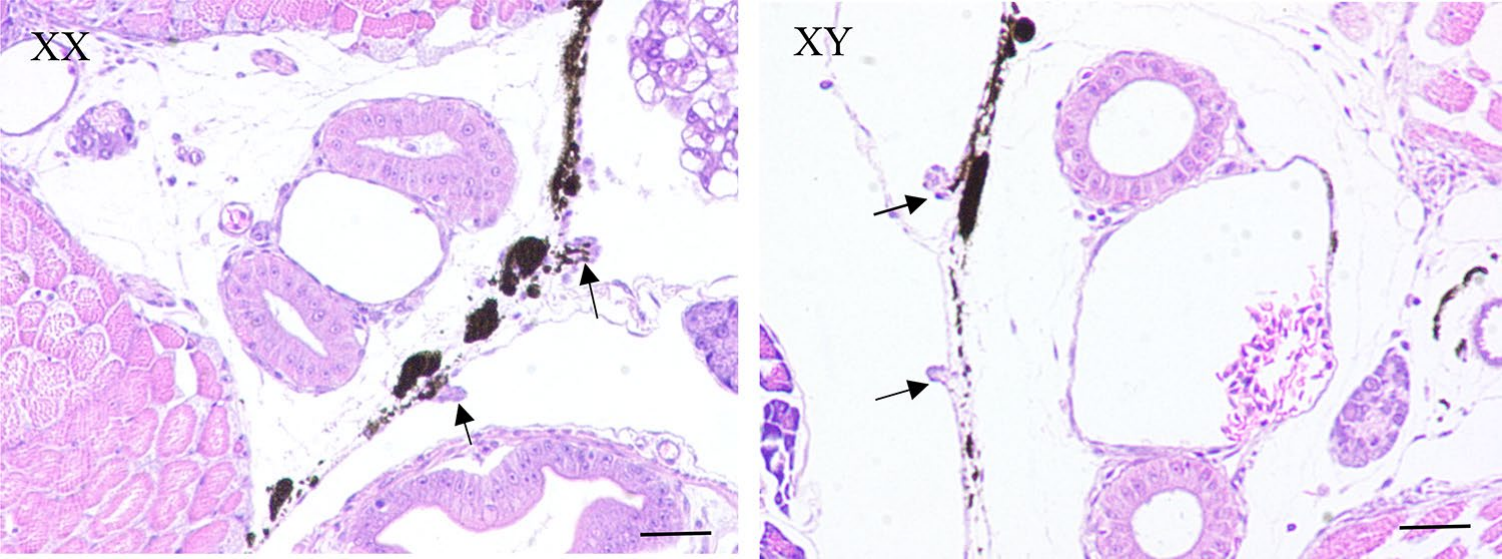
Hematoxylin–eosin stained sections of *T. rubripes* gonads from 40 days after hatching. Scale bars, 100 μm. Arrow, gonads.

**Figure 2:**
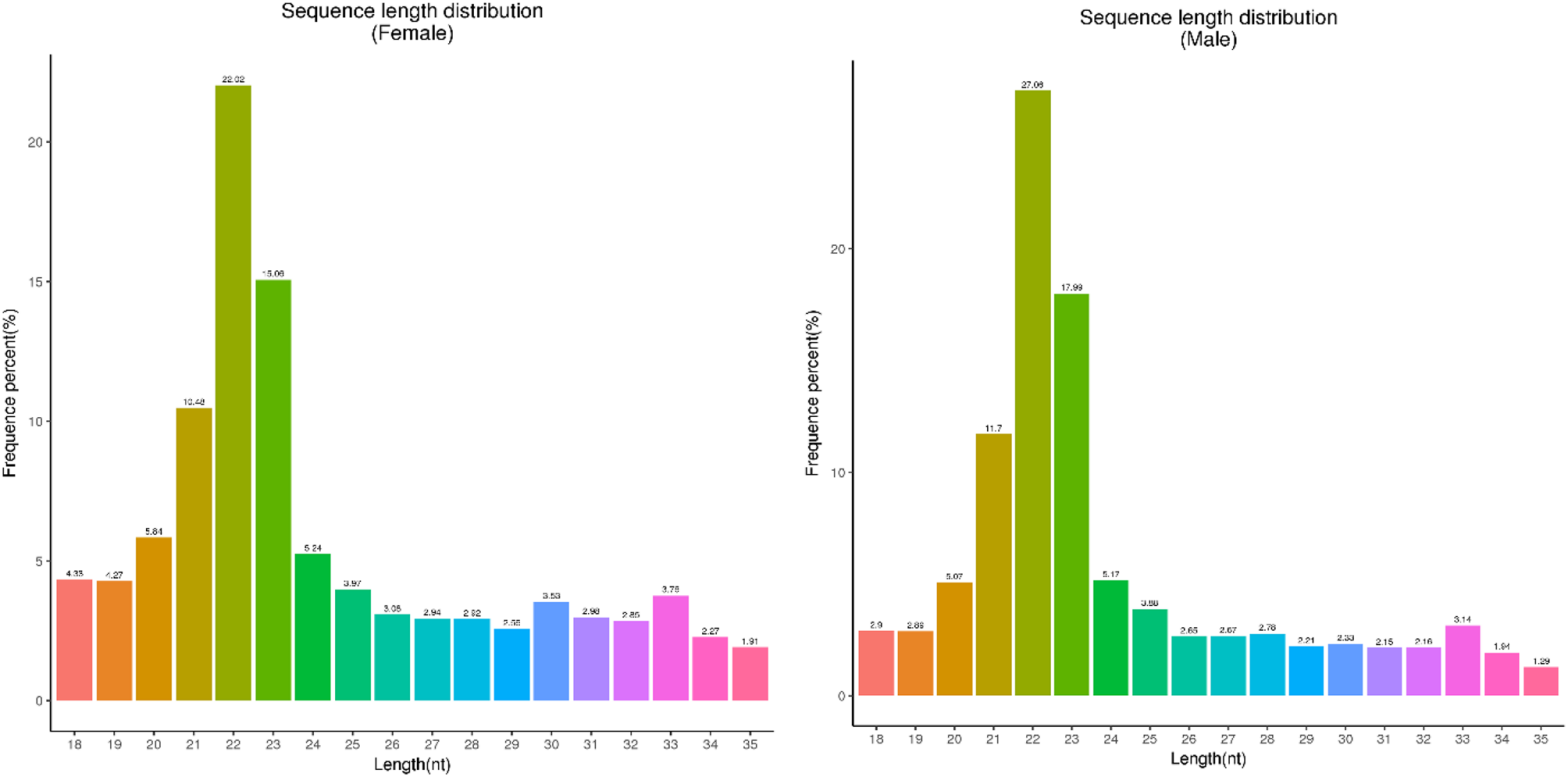
Length distribution of small RNA sequences in the XX (left) and XY (right) gonads of *T. rubripes*.

Gonadal sections of 40 days after hatching (dah) *T. rubripes* larvae confirmed that the characteristic of morphological sex differentiation was not observed in any of the specimens (Fig. 1). Based on the construction and sequencing of small RNA libraries, a total number of 16,653,352 raw reads was generated from female gonads and 19,117,245 from male gonads. After removing low-quality reads, adaptors, and contaminants, 13,151,941 (78.97%) clean reads from female gonads and 16,775,235 (87.75%) from male gonads were obtained. In total, 5,769,544 (57.69%) XX sequences and 9,720,240 (66.55%) XY sequences of the clean reads matched to the *T. rubripes* genome. Of these matched sequences, 2,193,958 (in XX) and 4,245,186 (in XY) matched miRNAs were found in the Rfam database (see Supplementary File S2). Size distribution analysis of small RNAs showed that 22 nt was the abundant size in both XX and XY gonads followed by 23 and 21 nt (Fig. 2).

A total of 107 known miRNAs and 97 novel miRNAs were expressed in XX fish, while 106 known miRNAs and 112 novel miRNAs were expressed in XY fish. In total, 107 conserved miRNAs and 124 novel miRNAs were identified in the gonads. The most abundant conserved miRNA was fru-miR-100, with 453,080 reads in female gonads and 906, 233 reads in male gonads. In addition, fru-miR-30d, fru-miR-181a-5p, fru-miR-21, fru-miR-22a, fru-miR-92, fru-miR-199, fru-miR-125a, fru-let-7d, fru-miR-25, and fru-miR-200b were found more than 100,000 times in male gonads, and fru-miR-92, fru-miR-30d, fru-miR-22a, fru-miR-21, and fru-miR-181a-5p were found more than 100,000 times in female gonads. Moreover, the expression of novel miRNAs was lower in *T. rubripes* gonads; novel_1 was the most abundant (52,989 reads in male gonads and 34,118 reads in female gonads (see Supplementary File S3).

### Target prediction and function annotation of miRNAs

A total of 12,517 target genes (12,464 were annotated) were predicted (see Supplementary File S4). Among them, fru-miR-15b was predicted to regulate the expression of *foxl2*, and novel-167, novel-318, and novel 538 were predicted to target to *dmrt1*. Some miRNAs target key genes related to TGF-β signaling; for example, *amh* was predicted to be the target of novel-548, *gata2* was predicted to be the target of novel-240, and *smad4* was predicted to be the target of novel-277, novel-407, and novel-512. Other miRNAs were predicted to target key genes in pathways involving sex steroid synthesis; for example, novel-358 and fru-miR-132 were predicted to target *star*, novel-451 was predicted to target *cyp17a12*, and fru-miR-15a, novel-496, fru-miR-122, and novel-538 were predicted to target *cyp11b*. Novel-128, fru-miR-187, and novel-542 were predicted to regulate the expression of *cyp11a1*.

**Figure 3:**
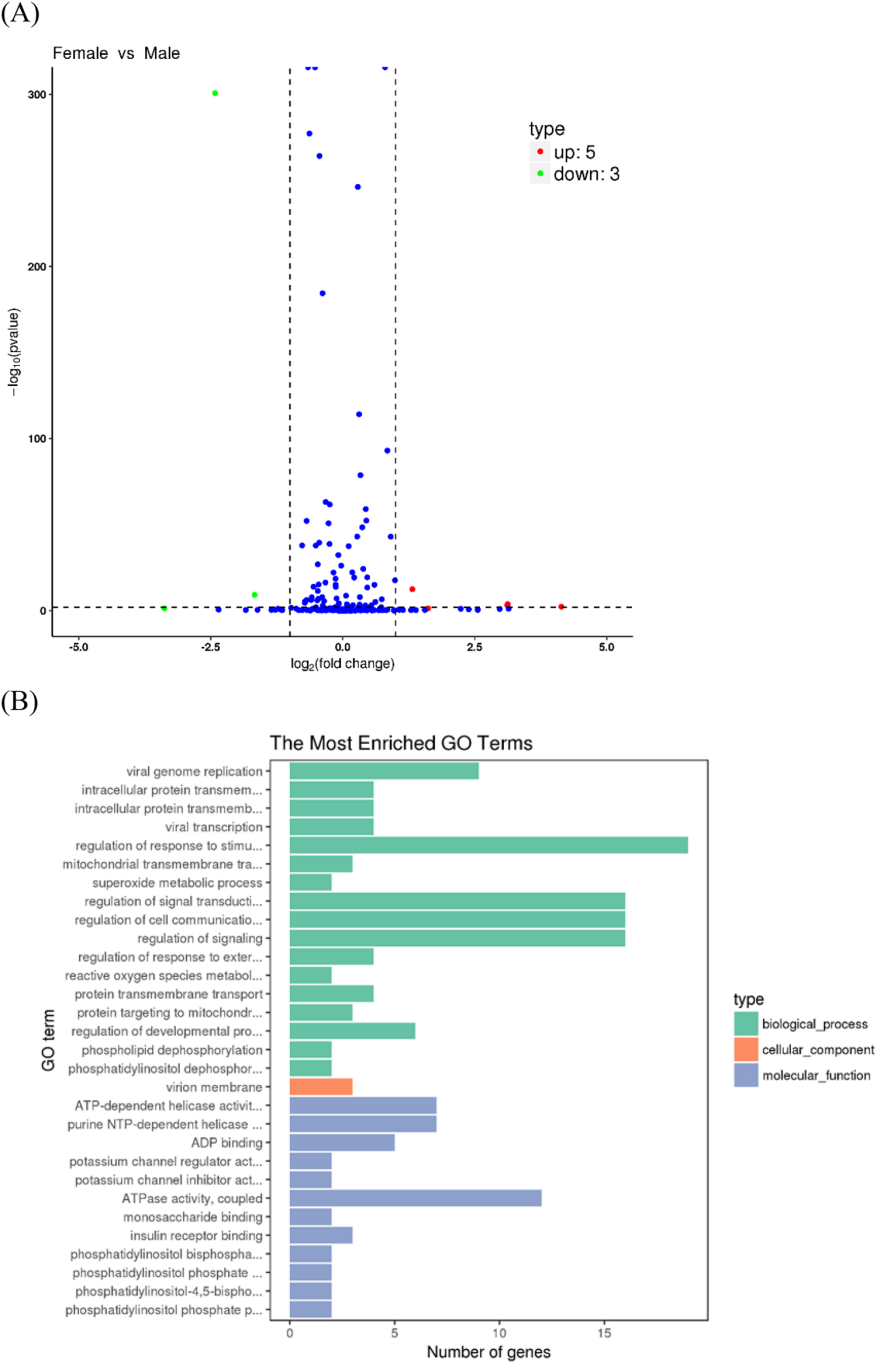
(**A**) Expression of miRNAs in the ovaries and testes of *T. rubripes*. The x-axis shows the fold change of expression levels between the XX and XY gonads. The y-axis shows the statistical significance of the change in miRNA expression. Blue dots indicate miRNAs that were equally expressed, red dots indicate upregulated miRNAs in XX gonads, and green dots indicate downregulated miRNAs in XX gonads. (**B**) Gene ontology (GO) analysis of the predicted target genes of the differentially expressed miRNAs. The GO enrichment of the predicted target genes in molecular functions, cellular components, and biological processes is shown.

**Figure 4:**
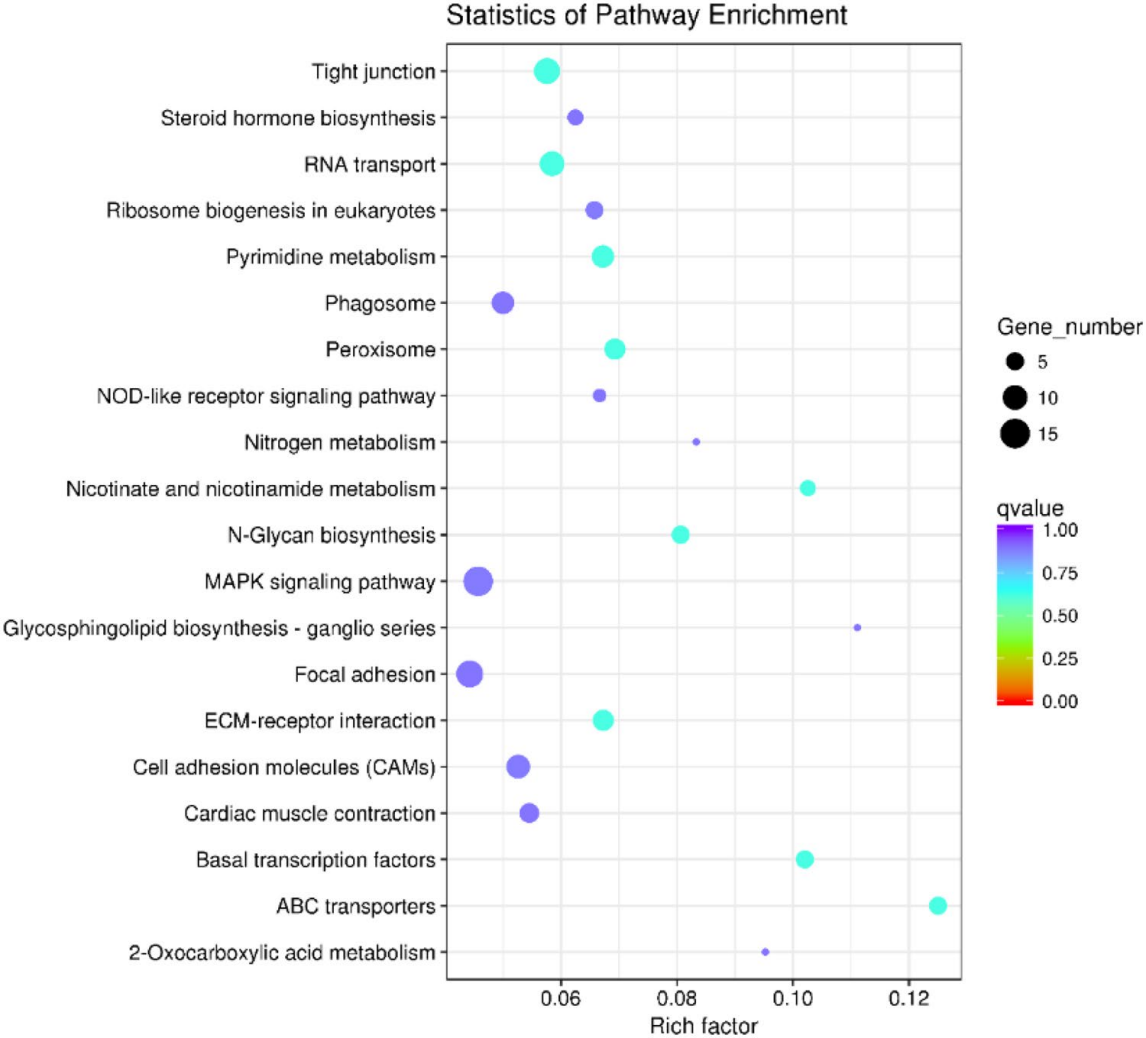
The 20 most enriched KEGG pathways enriched by the putative target genes of the differentially expressed miRNAs.

Five mature miRNAs were down-regulated and three mature miRNAs were up-regulated in male gonads compared with female gonads (Fig. 3A). In total, 1,076 potential target genes of the differentially expressed miRNAs were identified. Enrichment analysis of 631 target genes indicated that they were enriched in the biological process gene ontology (GO) terms regulation of response to stimulus (19), regulation of cell communication (16) and regulation of signal transduction (16), and regulation of signaling (16). At the molecular function level, the most enriched GO term was ATPase activity, coupled (12) (Fig. 3B). According to KEGG analysis, most of the target genes were enriched in MAPK signaling (15), tight junction (11), RNA transport (10), and focal adhesion (12) (Fig. 4).

### Overview of lncRNA sequencing

In total, 950,603,34 raw reads were produced and 922,149,64 clean reads were generated by removing reads mapped to rRNA and low-quality reads in female gonads. While in the male gonads, 857,388,18 raw reads were produced and 830,504,86 clean reads were generated. Of the clean reads, 75.66% and 66.67% were efficiently mapped against the *T. rubripes* reference genome in female and male libraries, respectively (see Supplementary File S5).

### Analysis of differentially expressed lncRNAs and their putative target genes

**Figure 5:**
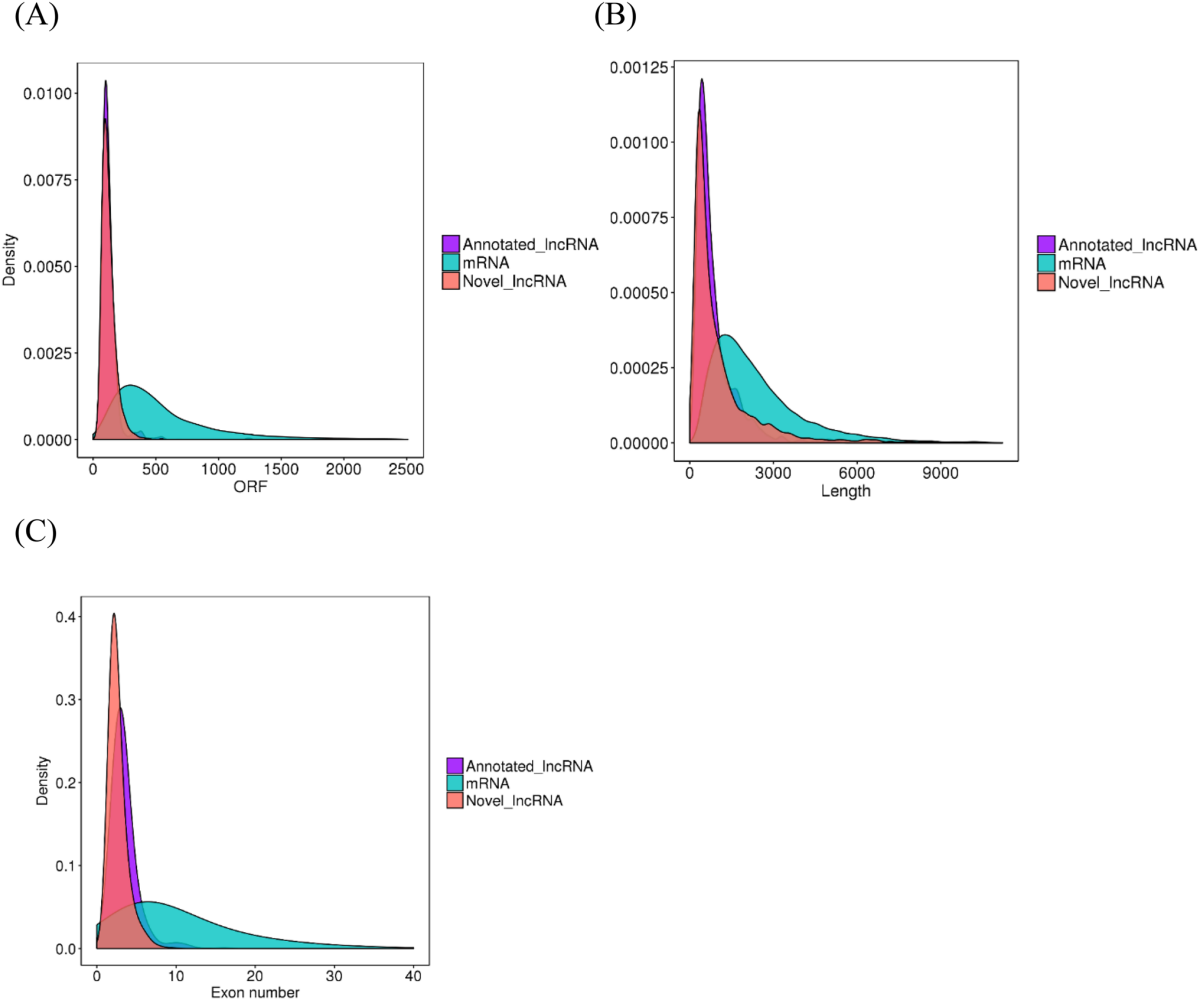
Comparison of features of predicted lncRNAs and mRNAs. (**A**) Expression of lncRNAs and mRNAs. (**B**) Length distribution of predicted lncRNAs and mRNAs. (**C**) Exon number distribution of lncRNAs and mRNAs.

Illumina RNA-seq analysis identified 31,362 mRNAs, including 30,187 known transcripts and 1175 putative transcripts. Of the remaining 2774 lncRNA transcripts, only 523 (18.85%) were previously identified, and 2251 (81.15%) were novel (see Supplementary File S6). The average mRNA length was 2732 bp (open reading frame, ORF, 573 bp) and the average length of lncRNAs was 2104 bp (ORF, 225 bp) in *T. rubripes* gonads (Fig. 5A,B). Moreover, both the annotated and novel mRNAs (12) contained, on average more exons than lncRNAs (5) (Fig. 5C).

**Figure 6:**
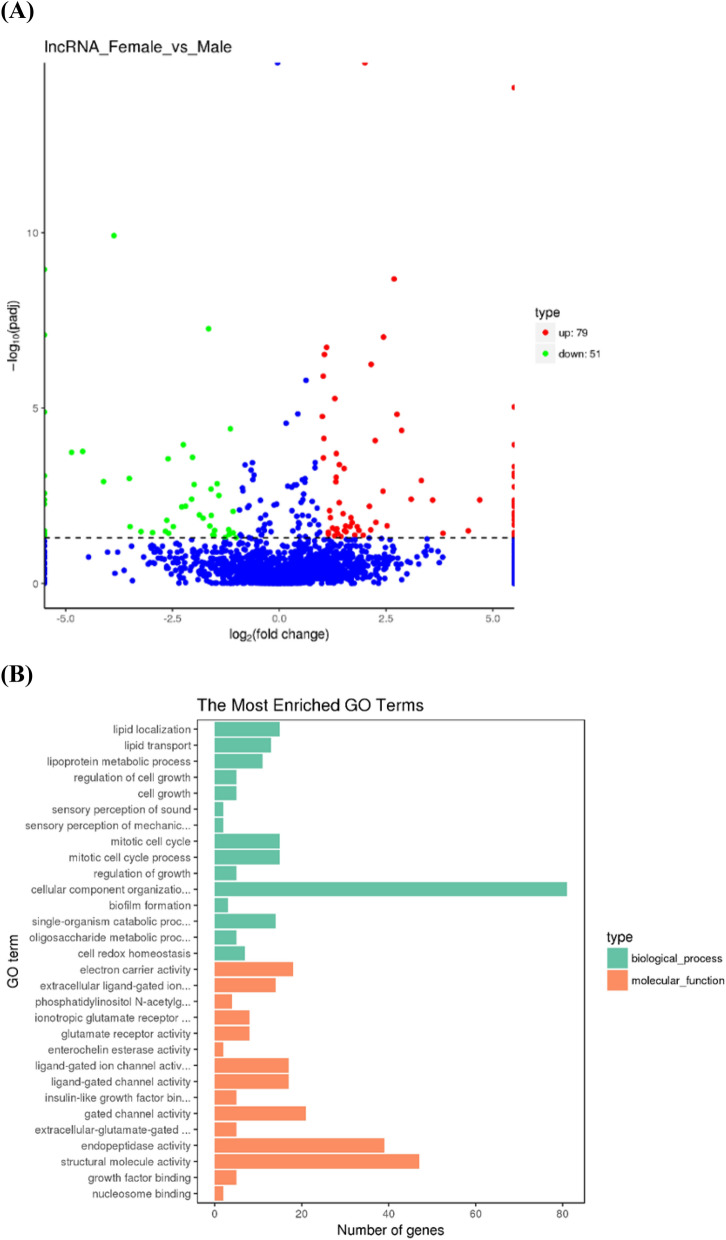
(**A**) Expression of lncRNAs in the ovaries and testes of *T. rubripes*. The x-axis shows the fold change of expression levels between the XX and XY gonads. The y-axis shows the statistical significance of the change in lncRNA expression levels. Blue dots indicate lncRNAs that were equally expressed, red dots indicate upregulated lncRNAs in XX gonads, and green dots indicate downregulated lncRNAs in XX gonads. (**B**) Gene ontology (GO) analysis of the predicted target genes of the differentially expressed lncRNAs. The GO enrichment of the predicted target genes in molecular functions, cellular components, and biological processes is shown.

A total of 130 lncRNA transcripts displayed sex-biased expression in *T. rubripes* gonads. Of which, 51 were down-regulated and 79 were up-regulated in male gonads compared with female gonads (Fig. 6A and Supplementary File S7). Overall, 2243 lncRNAs were predicted to target 13,093 genes (see Supplementary File S8). Interestingly, the predicted target genes of several sex-biased lncRNAs were sex-differentially expressed genes; these included lnc_000338, lnc_000370, lnc_000690, XLOC_021951, and XR_965485.1, which target *gsdf*, *thyroid hormone receptor-associated protein 3*, *thyroid hormone receptor beta*, *methyltransferase like protein*, and *zp3*, respectively. Enrichment analysis of 1122 target genes revealed that there were more target genes involved in cellular component organization (81) at the biological process level and structural molecule activity (47) and endopeptidase activity (39) at the molecular function level (Fig. 6B). KEGG analysis showed that the target genes were mainly enriched in neuroactive ligand-receptor interaction (24), calcium signaling (20), cGMP-PKG signaling (18), and Oxytocin signaling (17) (see Supplementary File S9).

### Validation of miRNAs and lncRNAs by qPCR

**Figure 7:**
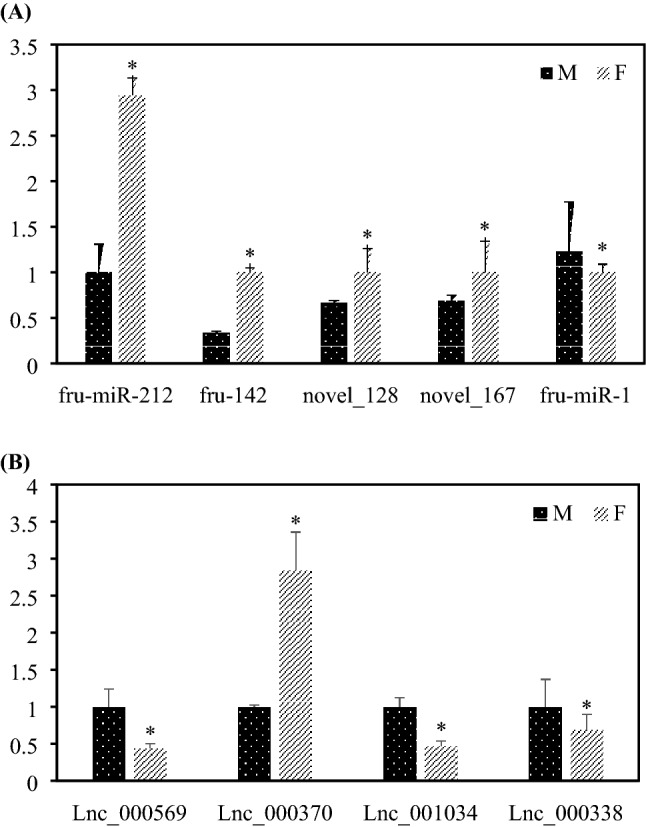
qPCR validation of the differentially expressed miRNAs (**A**) and lncRNAs (**B**) identified using Solexa sequencing. Each value represents the mean ± SEM of three measurements; *P < 0.05 between the XX and XY gonads, Student *t*-test.

The relative expression levels of five miRNAs (Fig. 7A) and four lncRNAs (Fig. 7B) from qPCR are shown in Fig. 7A. Fru-miR-212, fru-142, novel_128 and novel_167 were expressed at higher levels in XX than in XY, while fru-mir-1 was expressed at higher levels in XY than in XX at 40 dah (*p* < 0.05). The expression levels of Lnc_000569, Lnc_001034 and Lnc_000338 were significantly higher in XY, while the level of Lnc_000370 was significantly higher in XX (*p* < 0.05). The results are consistent with the sequencing data, demonstrating that the process used to identify putative miRNAs and lncRNAs was sufficiently stringent.

## Discussion

Recently, several studies have screened the sex-biased expressed miRNAs between female and male gonads, however, few studies have focused on the larvae at the early sex differentiation stages in teleosts^19,23^. Additionally, lncRNAs have been widely shown to play an essential role in the process of sex determination and differentiation in mammals, but their potential role in other vertebrates has only been reported in the Chinese soft-shell turtle^29^. In this work, the dominant size of small RNAs in both male and female gonads was 22 nt, followed by 23 and 21 nt, within the typical size range for Dicer-derived products. This phenomenon is similar to that in *O. niloticus*^19^and *C. carpio*^23^. Additionally, in another study of adult *T. rubripes*, the predominant size of small RNAs were 26–28 nt in the ovaries and testes, with most having a length of 27 nt^32^. In the gonads of *O. niloticus* and *D. rerio*, a difference in length distribution of small RNAs between adults and larvae has also been found^18,33^. This may be due to the higher expression of piRNAs in the gonads of those adult fish. PiRNAs are a class of 26–32 nt ncRNAs, which are expressed mainly in the germline and play a significant role in germline development.

In the present study, fru-miR-100, fru-miR-30d, fru-miR-181a-5p, fru-miR-21, fru-miR-22a, fru-miR-92, fru-miR-199, fru-miR-125a, fru-let-7d, fru-miR-25, and fru-miR-200b were the conserved miRNAs that were highly expressed in the gonads of *T. rubripes*. The results implied that these miRNAs probably play a critical and basal physiological role in the process of gonadal development in fugu. miR-100 is also abundant in the undifferentiated gonads of *O. niloticus*^19^, and in the mature ovaries and testes of *Pelteobagrus fulvidraco*, and *H. hippoglossus*^14,17^. In *T. ovatus*, the miR-30 family is highly expressed in the testes^34^. In *O. niloticus*^18^ and *D. rerio*^35^, the miR-181a family is also abundantly expressed. In *P. fulvidraco*, compared with XY, it was observed that miR-21-5p and miR-21-3p levels increased more than four fold in YY testes and XX ovaries^17^. Taken together, this indicated that the miR-100, miR-30, miR-181a, and miR-21 families play an essential role in the gonadal development of teleosts. The miR-17/92 cluster is a typical highly conserved and well-studied miRNA cluster, and has pivotal roles in the regulation of the cell cycle, proliferation, and apoptosis^36^. MiR-92 is abundant in the gonads of *T. rubripes*, similar to the results found in zebrafish (*D. rerio*) and yellow catfish (*P. fulvidraco*)^17,35,37^. A recent study has demonstrated that during the early stages of embryogenesis of *D. rerio*, maternal miR-92a-3p can suppress the cyclin dependent kinase 1 inhibitor (Wee 1 homolog 2), which regulates cell cycle progression. A significantly higher rate of embryonic developmental arrest at the one-cell embryo stage was observed due to the inhibition of maternal miR-92a-3p expression^38^. let-7 is one of the miRNAs family initially identified in *Caenorhabditis elegans* as a key developmental switch and plays a crucial role in controlling the transition from larva to adult^39^. Eight members of the let-7 family (let-7a/b/d/e/g/h/i/j) were identified in *T. rubripes* gonads in the present study. As a critical regulator of gene expression, let-7 miRNAs are abundant in gonadal tissues of mammals and are involved in multiple physiological processes, such as the regulation of sexual maturity, sperm formation and oocyte maturation^40–42^. In teleosts, the let-7 family was also highly expressed in the gonads of *Trachinotus ovatus*^34^, *C. carpio*^22^, *P. olivaceus*^23^, *Megalobrama amblycephala*^43^, *Danio rerio*^35^, and *Oryzias latipes*^25^, consistent with the expression in *T. rubripes*, indicating functional conservation across vertebrates and suggesting that the let-7 family might be crucial for reproductive physiology. In *P. olivaceus*, female-biased expression of miR-200b was found, and the expression level in the testes was sevenfold lower than in the ovaries^23^. In mice, it was shown that Dicer, a ribonuclease essential for miRNA biogenesis, is crucial for Sertoli cell maturation, survival, and ultimately sustenance of germ cell development^44^. Ablation of Dicer in Sertoli cells caused the loss of several miRNAs, including miR-125a-3p, and subsequent up-regulation of SOD-1, a protein linked to apoptosis^45^.

In the present study, five mature miRNAs were down-regulated and three mature miRNAs were up-regulated in undifferentiated XY gonads compared with XX gonads. We therefore selected five miRNAs for qPCR analysis, and Fru-miR-212, fru-142, novel_128 and novel_167 were expressed at higher levels in XX than in XY, while fru-mir-1 was expressed at higher levels in XY than in XX at 40 dah (*p* < 0.05). In another study of adult *T. rubripes*, some sexually dimorphic miRNAs were also found in gonads. For example, fru-miR-214, fru-miR-143-3p, fru-miR-202-5p, fru-miR-24-3p and fru-miR-145b-5p exhibited higher expression levels in the ovaries than in the testes, while fru-miR-2478-3p and fru-miR-2898-3p exhibited higher expression levels in the testes than in the ovaries^32^. The differentially expressed miRNAs in gonads are totally different in larval and adult fugu, which suggested that different miRNAs may be involved in the different gonadal developmental stages. Similar phenomena were observed in tilapia, for example, by transcriptomic analysis of gonads collected at five different time points (30, 50, 75, 100, and 165 days after fertilization). Xiao et al. found stage-specific expression patterns of miRNAs^18^. Tao et al. quantified the expression of miRNAs in the undifferentiated gonads at the critical stage of molecular sex determination (5 dah), and obtained many different sex-biased expression miRNAs which different from tnat obtained in Xiao et al.^18,19^. Since limited reports focus on the different stage-specific expression levels, especially in teleosts, more work should be performed to elucidate the function miRNAs during sex differentiation and gonadal development, and the underlying mechanisms. Target prediction suggested that some miRNAs target key genes that participate in the process of sex differentiation of *T. rubripes*. Notably, novel-167 is enriched in the gonads of XX *T. rubripes*, and *dmrt1* was its candidate direct target gene. In vertebrates, several DM domain genes (*dmrt* genes) have been shown to be required for gametogenesis and gonadal differentiation. *Dmrt1* seems to have a more significant role and likely regulates testicular differentiation in all vertebrates^46^. Our previous study reported that XY *T. rubripes* have higher expression levels of *dmrt1* in undifferentiated gonads compared with XX individuals^31^. During the early life stages of *T. rubripes*, 17-beta estradiol treatment was able to induce decrease of *dmrt1* levels and feminization in XY individuals^47,48^. Therefore, *dmrt1* may play a pivotal role during *T. rubripes* testicular differentiation, as reported in other species, and novel-167 is possibly involved in the regulation of *dmrt1* transcription in *T. rubripes*.

Similar to previous studies in other vertebrates, the identified *T. rubripes* lncRNAs had shorter transcripts, lower expressions level, and fewer exons than the identified mRNAs^49^. *T. rubripes* lncRNAs (average, 2104 nt) were shorter than those in chicken (2941 nt)^50^, but longer than those in mouse (550 nt), human (1000 nt), zebrafish (1113 nt), goat (1180 nt), and Chinese soft-shell turtle (1717 nt)^29,51–53^. Additionally, on average, lncRNA transcripts in *T. rubripes* contained more exons than those in mouse (3.7), human (2.9), zebrafish (2.8), goat (2.2), and Chinese soft-shell turtle (2.0)^29,51–53^.

This study aimed to identify lnRANs involved in the onset of sexual differentiation in *T. rubripes*. We found that 130 out of 2774 lncRNAs were differentially expressed in male and female gonads. Among them, four lncRNAs were randomly selected for qPCR, and the qPCR results were consistent with the sequencing data. Moreover, some sex-differentially expressed genes are predicted to be the target of sex-biased lncRNAs, indicating these lncRNA-gene pairs may play an important role in fugu sexual differentiation. Remarkably, a new member of the TGF-β superfamily, *gsdf* was found to be a potential target of lncRNA000338*. gsdf* restricts expression in gonadal somatic cells in teleosts and gain- and loss-of-functional analysis demonstrated it is essential for teleost sex determination and differentiation^54,55^. In the male undifferentiated gonads of *T. rubripes*, higher expression levels of *gsdf* were observed, suggesting that it may have a similar role as described in other telosts^31^. Further study should be focused on validating the functional analysis of *gsdf* and lncRNAs, and the relationships and regulatory mechanisms between them.

## Materials and methods

### Samples collection, RNA extraction, and histological observation of gonads

Eighty larvae with 1.92 ± 0.19 cm average full length at 40 dah were obtained from a fishery in Dalian, China in April 2018. Then, 60 T*. rubripes* were anesthetized, and the gonads were dissected, flash-frozen in liquid nitrogen and stored at − 80 °C. Since they are tiny, the gonads of each individual were placed into a tube one by one. For sex verification of each larva, a piece of tissue sample was stored in a 1.5 ml tube containing 100% alcohol in a freezer at − 20 °C. After sex verification, gonads with the same gender (20 gonads/sex) were pooled, and RNA was extracted from male and female fish as described in our previous study^31^. Briefly, DNA was extracted from the tissues according to the manufacturer’s protocol for the TIANamp Marine Animals DNA kit (Tiangen, Beijing, China), and the genetic sex of each fish was identified using SNP markers (a region containing exon 9 of the *amhr2* gene). As shown in Supplementary File S1, the genotype of males was C/G (XY) and that of females was C/C (XX). Previous studies have demonstrated there is a perfect concordance between the SNP genotype and phenotypic sex^30,31,56^. Histological observation of gonads was also conducted as described in our previous study^31^. All experiments with fugu were approved by the Animal Study Ethical Committee of Fishery Resources Enhancement Laboratory at Dalian Ocean University (Dalian, China). Experiments were performed in accordance with the lab animal protection regulations and guidelines of the People’s Republic of China (Order of the State Council of the People’s Republic of China No. 676), Liaoning Province (Order No. 143 of the people’s government of Liaoning province).

### Library preparation and sequencing

RNA purity and concentration were examined as described in our previous study^31^. The small RNA libraries were prepared using an RNA-Seq library preparation kit (NEBNext Multiplex Small RNA Library Prep Set for Illumina, NEB, USA.) following the manufacturer’s recommended instructions. After quality certification and the cluster generation, two transcriptome libraries were sequenced on an Illumina Hiseq 2500 platform.

For lncRNA library construction, rRNA was firstly removed (Epicentre Ribo-zero rRNA Removal Kit, Epicentre, USA). After the rRNA-depleted RNA fragmentation, two sequencing libraries were generated according to the manufacturer’s protocol using the NEBNext Ultra Directional RNA Library Prep Kit for Illumina (NEB, USA). Subsequently, the library fragments were purified using an AMPure XP system (Beckman Coulter, Beverly, USA) to preferentially select cDNA fragments of 150–200 bp, and then the libraries sequencing was carried out on an Illumina Hiseq 4000 platform (paired-end 150 bp reads).

### Small RNA sequence analysis

Data processing was carried out according to previously described procedures^34^. Clean reads were obtained by removing adapter sequences, low-quality sequences, sequences containing poly-N, and tags originating from protein coding genes, repeat sequences, rRNA, tRNA, snRNA, and snoRNA. Then, reads between 18 and 32 nt in length were selected for mapping to the *T. rubripes* genome using Bowtie, and the mapped small RNA tags were used to look for known miRNAs. The MiRBase20.0 (ftp://mirbase.org/pub/mirbase/20/) was used as reference, and modified software programs mirdeep2 and sRNA-tools-cli were used to obtain the potential miRNAs and draw the secondary structures. Two software programs, miREvo and mirdeep2, were integrated to predict novel miRNAs. miFam.dat (http://www.mirbase.org/ftp.shtml) was used to look for families for conserved miRNAs, while Rfam (http://rfam.sanger.ac.uk/search/) was used to look for Rfam families for novel miRNA.

### Transcriptome (mRNA) sequence analysis and identification of candidate lncRNAs

Clean data were obtained by removing low-quality reads, reads containing adapters, and reads containing poly-N from raw data. Bowtie2 v2.2.8 was used to build the index of the reference genome, and HISAT2 v2.0.4 was used to align the clean reads to the reference genome (ftp://ftp.ncbi.nlm.nih.gov/genomes/all/GCF_000180615.1_FUGU5). The lncRNA transcriptome was assembled using StringTie (v1.3.1) in a reference-based approach^57^.

Three types of coding potential analysis softwares, coding-Non-Coding-Index, coding Potential Calculator, and Pfam Scan (v1.3) were combined to screen the non-protein coding RNA candidates from putative mRNAs. The phylogenetic codon substitution frequency (PhyloCSF) metric (v20121028) was used to build the multi-species genome sequence alignments with default parameters. After the above analysis, transcripts without coding potential predicted by either/all of the four tools were candidate lncRNAs.

### Prediction and annotation of miRNA and lncRNA targets

Predictions of target genes of miRNAs was analyzed by miRanda. Since lncRNA can cis target their neighboring genes, the coding genes form 10 kb downstream and upstream of all identified lncRNAs were searched and their functions were analyzed. Pearson’s correlation coefficients between expression levels of lncRNAs and mRNAs were calculated with custom scripts^58^.

### Differential expression of identified miRNAs and lncRNA, and enrichment analysis of their target genes

In order to compare the differential expression of miRNA in female and male gonads, the expression levels of miRNAs were calculated by the transcript per million (TPM) approach firstly^34^. Then, based on TPM normalized counts, the analysis was carried out using the DEGseq (2010) R package. The criteria used for screening the differentially expressed miRNAs were *q*-value < 0.05 and |log2(fold change)| > 1.

Cuffdiff (v2.1.1) was used to calculate fragments per kilobase of transcript per million mapped reads values for both lncRNAs and mRNAs in each sample^59^. The Ballgown suite includes functions for interactive exploration of the transcriptome assembly, visualization of transcript structures and feature-specific abundances for each locus, and post-hoc annotation of assembled features to annotated features^60^. Transcripts with an adjusted *P*-value < 0.05 and |log_2_(fold change)| > 1 were assigned as differentially expressed.

GOseq-based Wallenius non-central hyper-geometric distribution and KOBAS software were used for GO and KEGG pathway enrichment analysis of the putative target genes^61,62^. GO terms or KEGG pathway with corrected *P* < 0.05 were considered significantly enriched in differentially expressed genes.

### Validation of sex-biased miRNAs and lncRNAs

Five miRNAs and four lncRNAs were randomly chosen for qPCR validation according to previously described methods^31^. The expression of miRNAs and lncRNAs were determined with a LightCycler480II Real-Time PCR System, in which *U6* and β*-actin* were used as reference genes, respectively. The sequences of primers, including miRNA-specific stem-loop RT primers are listed in Table 1. All Reactions were carried out in triplicate, and the relative expression of the selected miRNAs and lncRNAs was calculated by the 2^−ΔΔCT^ method. The Student *t*-test was conducted to evaluate the statistical significance between female and male gonads. Statistical analysis was carried out using SPSS version 22.0 software (IBM, Chicago, IL, USA). A *p*-value of < 0.05 was deemed to be significant.

**Table 1:** Primers used for qPCR in the present study.

Primer name	Primer sequence (5′–3′)	Product size (bp)
U6-F	CTCGCTTCGGCAGCACATATACT	93
U6-R	ACGCTTCACGAATTTGCGTGTC	
novel_128-F	GATCATCCGCCCTAACTCC	69
novel_128-R	TCGTATCCAGTGCAGGGTC	
novel_128-Stem loop	GTCGTATCCAGTGCAGGGTCCGAGGTATTCGCACTGGATACGAAAGCAGAG	51
novel-167-F	CGTCGAGAGCAGGATCAGT	69
novel-167-R	TCGTATCCAGTGCAGGGTC	
novel-167-Stem loop	GTCGTATCCAGTGCAGGGTCCGAGGTATTCGCACTGGATACGACCCGATCC	51
fru-miR-212-F	TGCGTGCCTAACAGTCTACAG	71
fru-miR-212-R	TCGTATCCAGTGCAGGGTC	
fru-miR-212-Stem loop	GTCGTATCCAGTGCAGGGTCCGAGGTATTCGCACTGGATACGAAGCCATGA	51
fru-miR-142-F	CGGTCGTGCATAAAGTAGAAA	71
fru-miR-142-R	TCGTATCCAGTGCAGGGTC	
fru-miR-142-Stem loop	GTCGTATCCAGTGCAGGGTCCGAGGTATTCGCACTGGATACGAAGTAGTGC	51
fru-miR-1–F	CCGACGTGGAATGTAAAGAA	70
fru-miR-1–R	TCGTATCCAGTGCAGGGTC	
fru-miR-1–Stem loop	GTCGTATCCAGTGCAGGGTCCGAGGTATTCGCACTGGATACGAATACATAC	51
β-actin–F	CAATGGATCCGGTATGTGC	245
β-actin–R	CGTTGTAGAAGGTGTGATGCC	
LNC_000338-F	TGCCTCAGACATCTCTGACAGG	225
LNC_000338-R	CTAGTGCTGGCTTGTGTCATCC	
LNC_000569-F	GGATGCCAGGGAATAGAGAAG	147
LNC_000569-R	TTCTTTGTTGACGGCAGTCAC	
LNC_000370-F	ACAGCGTCAAGTACACAAAGCC	153
LNC_000370-R	TTAGGAGATCGGAAGGGACAGT	
LNC_001034-F	CGTCAGAGGTGGATTTGCATAC	145
LNC_001034-R	ACGTGCATCGACATAGACGAAT	

## Supplementary Information

Supplementary Legends.

Supplementary File S1.

Supplementary File S2.

Supplementary File S3.

Supplementary File S4.

Supplementary File S5.

Supplementary File S6.

Supplementary File S7.

Supplementary File S8.

Supplementary File S9-1.

Supplementary File S9-2.

## Data Availability

The Illumina sequences data of this study have been submitted to the Short Read Archive of the National Center for Biotechnology Information (NCBI) (Accession Numbers: PRJNA646544, PRJNA646995 and PRJNA647059).
