# Supplementary material for: Identification of sex differentiation-related microRNA and long non-coding RNA in Takifugu rubripes gonads
Source: Sci Rep. 2021 Apr 2;11:7459. doi: 10.1038/s41598-021-83891-w (PMC8018949; doi:10.1038/s41598-021-83891-w)
Supplement: Supplementary file 1 — Supplementary Legends. [file 41598_2021_83891_MOESM1_ESM.docx]

**Identification of sex differentiation-related microRNA and lncRNA in *Takifugu rubripes* gonad**

**Hongwei Yan^1,2^, Qi Liu^1,2^, Jieming Jiang^1,2^, Xufang Shen^1,2^, Lei Zhang^1,2^, Zhen Yuan^1,2^, Yumeng Wu^1,2^ & Ying Liu^1,2*^**

^1^Dalian Ocean University, Dalian, Liaoning 116023, China.^2^Key Laboratory of Environment Controlled Aquaculture, Ministry of Education, Dalian, 116023, China. Hongwei Yan and Qi Liu contributed equally to this work. *, Corresponding author, email: 13953208976@163.com (Y. Liu).

**Supplementary information**

**Supplementary File S1.** Differential amhr2 chromatograms from male and female *Takifugu rubripes*. All of the males are heterozygous and the females are homozygous at the sex-associated SNP in amhr2 sequence.

**Supplementary File S2.** Reads produced by the Illumina HiSeq2500 platform.

**Supplementary File S3.** The TPM of expressed miRNAs in fugu gonads.

**Supplementary File S4.** The predicted target genes of miRNAs.

**Supplementary File S5.** Reads produced by the Illumina HiSeq4000 platform.

**Supplementary File S6.** Identified mRNAs and lncRNAs by the Illumina HiSeq4000 platform.

**Supplementary File S7.** Dysregulated sex differentiation-related lncRNAs.

**Supplementary File S8.** The protein coding genes as potential targets in the regions located

10-kb upstream and downstream of the lncRNAs.

**Supplementary File S9.** The 20 most enriched KEGG pathways enriched by the putative target genes of the diﬀerentially expressed lncRNAs.
