# Supplementary figures and images for: Identification of sex differentiation-related microRNA and long non-coding RNA in Takifugu rubripes gonads
Source: Sci Rep. 2021 Apr 2;11:7459. doi: 10.1038/s41598-021-83891-w (PMC8018949; doi:10.1038/s41598-021-83891-w)

Supplementary File S1


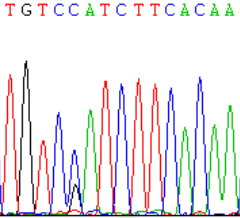


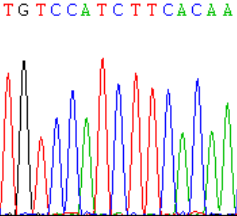


male female

Supplement: Supplementary file 2 — Supplementary File S1. [file 41598_2021_83891_MOESM2_ESM.docx]

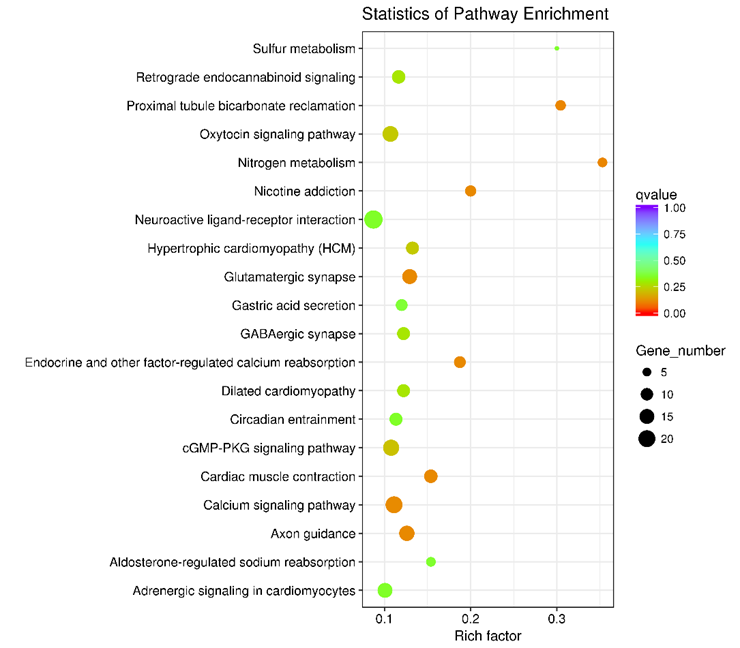
Supplementary File S9

Supplement: Supplementary file 10 — Supplementary File S9-1. [file 41598_2021_83891_MOESM10_ESM.docx]
